# To Solder Steel

**Published:** 1888-11

**Authors:** 


To Solder Steel.—As the pieces of steel become of a suitable temperature to be perfectly and easily soldered together after seeing they are treated and covered with a solution of silicate of soda, or an other solution with a base of silex—Mr. Middleton works thus: On plunging the pieces to solder in the silicate, well coating them with this material; then hold them in a packet at an ordinary soldering temperature. After this operation the pieces are passed under the soldering cylinders which allow them to obtain a perfect finish.—*Revue Scientifique.*

